# Brainstem–cortex disconnection in amyotrophic lateral sclerosis: bulbar impairment, genotype associations, asymptomatic changes and biomarker opportunities

**DOI:** 10.1007/s00415-023-11682-6

**Published:** 2023-04-06

**Authors:** Marlene Tahedl, Ee Ling Tan, Rangariroyashe H. Chipika, Jennifer C. Hengeveld, Alice Vajda, Mark A. Doherty, Russell L. McLaughlin, We Fong Siah, Orla Hardiman, Peter Bede

**Affiliations:** 1 Computational Neuroimaging Group (CNG), Trinity Biomedical Sciences Institute, Trinity College Dublin, Room 5.43, Pearse Street, Dublin 2, Dublin, Ireland; 2 Smurfit Institute of Genetics, Trinity College Dublin, Dublin, Ireland; 3 Department of Neurology, St James’s Hospital, Dublin, Ireland

**Keywords:** Amyotrophic lateral sclerosis, Biomarkers, Bulbar dysfunction, Motor neuron disease, Neuroimaging, Clinical trials, MRI

## Abstract

**Background:**

Bulbar dysfunction is a cardinal feature of ALS with important quality of life and management implications. The objective of this study is the longitudinal evaluation of a large panel imaging metrics pertaining to bulbar dysfunction, encompassing cortical measures, structural and functional cortico-medullary connectivity indices and brainstem metrics.

**Methods:**

A standardised, multimodal imaging protocol was implemented with clinical and genetic profiling to systematically appraise the biomarker potential of specific metrics. A total of 198 patients with ALS and 108 healthy controls were included.

**Results:**

Longitudinal analyses revealed progressive structural and functional disconnection between the motor cortex and the brainstem over time. Cortical thickness reduction was an early feature on cross-sectional analyses with limited further progression on longitudinal follow-up. Receiver operating characteristic analyses of the panel of MR metrics confirmed the discriminatory potential of bulbar imaging measures between patients and controls and area-under-the-curve values increased significantly on longitudinal follow-up. *C9orf72* carriers exhibited lower brainstem volumes, lower cortico-medullary structural connectivity and faster cortical thinning. Sporadic patients without bulbar symptoms, already exhibit significant brainstem and cortico-medullary connectivity alterations.

**Discussion:**

Our results indicate that ALS is associated with multi-level integrity change from cortex to brainstem. The demonstration of significant corticobulbar alterations in patients without bulbar symptoms confirms considerable presymptomatic disease burden in sporadic ALS. The systematic assessment of radiological measures in a single-centre academic study helps to appraise the diagnostic and monitoring utility of specific measures for future clinical and clinical trial applications.

**Supplementary Information:**

The online version contains supplementary material available at 10.1007/s00415-023-11682-6.

## Introduction

Two of the key barriers to the development of effective disease-modifying therapies in amyotrophic lateral sclerosis (ALS) are the late inclusion into pharmacological trials, and the lack of validated quantitative monitoring markers. Bulbar presentation in ALS has been consistently associated with shorter survival, faster functional decline and increased multidisciplinary support needs. Dysarthria has considerable quality of life implications and may impact on employment, social interactions and mood. Dysphagia may lead to weight loss, malnutrition, dehydration, aspiration pneumonia, sialorrhoea and increased risk for hospital admissions. Pseudobulbar affect may be misinterpreted as depression or behavioural change in the community, and may lead to social isolation. Despite these sombre sequelae, the substrate of bulbar impairment in ALS is relatively understudied radiologically, and proxies of bulbar impairment are also underrepresented among clinical trial outcome measures [1]. Imaging studies in ALS overwhelmingly focus on cortical atrophy and corticospinal tract changes even though brainstem and corticobulbar tract degeneration are hallmark pathological features of ALS and have been associated with the condition since its earliest descriptions. In one of the first pathologically supported reports in 1867, Lockhart Clarke eloquently describes progressive bulbar involvement in ALS: “Her voice changed; she did not pronounce words as usual…Her deglutition now became difficult… The tongue is atrophied on each side, and in folds, reminding one of cerebral convolutions. Her talking is nearly unintelligible” [2]. This moving description from over 150 years ago elegantly illustrates bulbar impairment in ALS which continues to affect patients today. Despite historical descriptions of brainstem atrophy and corticobulbar tract degeneration, these structures remain notoriously understudied in vivo. Brainstem pathology is regarded as ‘stage 1’ of a recently proposed four-stage pathological staging system based on pathological TDP-43 burden patterns [3], a staging-scheme increasing supported by radiological data [4]. Brainstem pathology is not unique to ALS, it is a shared feature of several motor neuron diseases [5], preferentially affecting the descending pyramidal tracts, cranial nerve nuclei or both. So, while corticobulbar tract and brainstem pathology are ‘disease-defining’ features of ALS with dramatic clinical ramifications, they are seldom evaluated systematically from cortex to brainstem in large multimodal longitudinal imaging studies. Accordingly, the main objective of this study is the evaluation of a comprehensive panel of cortical, brainstem and cortex-brainstem connectivity metrics to appraise their longitudinal trajectory, discriminatory power and association with relevant clinical metrics. An additional objective is the characterisation of bulbar integrity and corticobulbar connectivity in patients carrying the GGGGCC hexanucleotide expansion in *C9orf72*. Moreover, as existing presymptomatic studies exclusively assess radiological changes in gene carriers, we specifically evaluate the radiological profile of sporadic patients with spinal onset disease who are asymptomatic from a bulbar perspective at the time of their scan to estimate bulbar and corticobulbar disease-burden prior to symptom manifestation. Our main hypothesis is that a panel of structural and functional MR metrics may capture progressive cortico-medullary disconnection. We also hypothesise larger disease burden in *C9orf72* carriers and some degree of presymptomatic change in patients without bulbar disability.

## Methods

### Participants

Imaging data from 198 patients with ALS and 108 healthy controls (HC) were included in this study (Table 1). All participants gave informed consent in accordance with the Ethics Approval of this research project (Beaumont Hospital, Dublin, Ireland – IRB REC08/90). A prospective study design was implemented with the recruitment of incidence ALS cases. The mean symptom duration of patients from symptom onset to first scan was 15.5 months (median 16) with a range of 5–27 months. Participating ALS patients were diagnosed according to the revised El Escorial criteria. Exclusion criteria included prior neurosurgery, prior cerebrovascular events, traumatic brain injury, comorbid neoplastic, or neuroinflammatory diagnoses. Patients with comorbid psychiatric disease, patients who were unable to tolerate MR scanning, patients with incomplete MR acquisition and patients without genetic information were excluded. Inter-scan interval for longitudinal follow-up was four months. In three main analysis streams, patients were either assessed as a single group (*analysis stream 1*), stratified by *C9orf72* status (*analysis stream 2*) or by the presence bulbar symptoms (*analysis stream 3*). Basic demographic and clinical variables (age, sex, handedness, years of education, medications, body region of symptom onset, family history of ALS or FTD), were recorded on the day of MRI scanning and all patients had their total ALSFRS-r and ALSFRS-r sub-scores documented at the time of their scan. All patients were screened for intronic GGGGCC repeat expansion in *C9orf72* by repeat-primed PCR. Capillary electrophoresis outcomes were visualised using GeneMapper version 4.0 and patients exhibiting 30 or more repeats were considered *C9orf72*-positive. Additionally, all participating patients were screened for a panel of protein-altering, exonic or splice-site variants present in 32 genes linked to ALS in the ALS online database (ALSod). [6]

**Table 1:** The demographic and clinical profile of study participants

	All ALS patients	HC	Welch two-sample *t*-test [W] or Chi-squared [C2] [ALS vs. HC]	C9 +	C9–	BA (C9–)	BS (C9–)
Total number of subjects	198	108	*n.a*	22	176	78	98
Age [y, mean ± SD]	59.78 ± 11.98	59.01 ± 10.72	*W: t*(241.6) = – 0.58, *p* = 0.57	55.77 ± 8.45	60.29 ± 12.28	58.64 ± 12.84	61.60 ± 11.72
Sex, F/M	69/129	54/54	*C2: X*^*2*^ (1, *N* = 306) = 6.06, *p* = 0.01*	9/13	60/116	23/55	37/61
Handedness, R/L	182/16	101/7	*C2: X*^*2*^ (1, *N* = 306) = 0.08, *p* = 0.78	19/3	163/13	72/6	91/7
Years of education [y, mean ± SD]	13.56 ± 3.45	14.69 ± 3.55	*W: t*(215.07) = 2.67, *p* = 0.008*	13.73 ± 3.47	13.54 ± 3.46	13.55 ± 3.42	13.53 ± 3.51
ALSFRSR-score ± SD (baseline)	37.97 ± 7.18	*n.a*	*n.a*	31.68 ± 11.77	38.76 ± 5.98	40.68 ± 4.88	37.22 ± 6.35
Baseline scans [count]	198	108	*n.a*	22	176	78	98
4-month follow-up [count]	107	18	*n.a*	7	100	48	52
8-month follow-up [count]	65	13	*n.a*	7	58	26	32
12-month follow-up [count]	34	8	*n.a*	3	31	12	19
16-month follow-up [count]	2	0	*n.a*	0	2	2	0

*ALS* amyotrophic lateral sclerosis, *ALSFRS-r* revised ALS functional rating scale, *BA* bulbar asymptomatic, defined as *C9orf72* negative with spinal onset disease an no bulbar symptom at baseline, *BS* bulbar symptomatic, defined as *C9orf72* negative patient with bulbar symptoms at baseline, *C9 +*: hexanucleotide repeat expansion carrier in *C9orf72*, *C9-* tested negative for hexanucleotide repeat expansions in *C9orf72*, *F* female, *HC* healthy control, *L* left-handed, *M* male, *MRI* magnetic resonance imaging, *N* sample size, *n.a.* not applicable, *R* right-handed, *SD* standard deviation, *y* years, *significant at an alpha-level of *p* <  = 0.05

### MR imaging

**Fig. 1:**
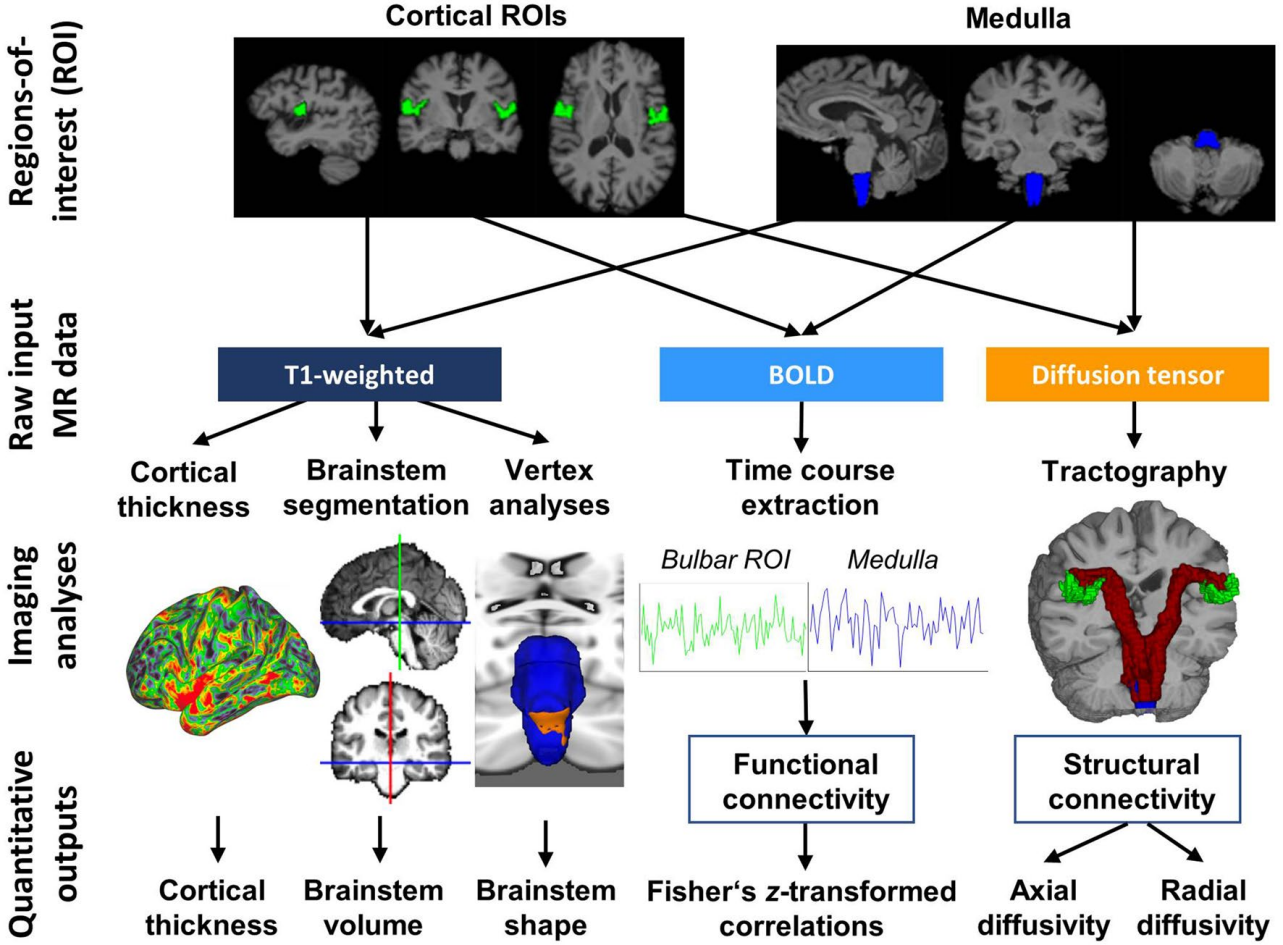
The workflow of data analyses from raw data to anatomical integrity metrics. A panel of cortical, brainstem, structural and functional cortico-medullary imaging measures were evaluated in a prospective, single-centre, longitudinal study. *AD* axial diffusivity, *ALS* patients with amyotrophic lateral sclerosis, *BOLD* blood-oxygen-level-dependent signal, *CT* cortical thickness, *DTI* diffusion tensor imaging, *DWI* Diffusion-weighted imaging, *FC* functional connectivity, *fMRI* functional MRI, *HC* Healthy control, *RD* radial diffusivity, *ROI* Region of interest

MR data were acquired on a 3 Tesla Philips Achieva platform and the protocol included structural T1-weighted (T1w), resting-state functional MR (rsfMRI) and diffusion-weighted (DWI) pulse-sequences (Fig. 1). The imaging protocol has been previously described [7]. Briefly, T1-weighted (T1w) images were acquired with a 3D Inversion Recovery prepared Spoiled Gradient Recalled echo (IR-SPGR) sequence with the following parameters; field-of-view (FOV) of 256 × 256 × 160 mm, flip angle = 8°, spatial resolution of 1 mm3, SENSE factor = 1.5, TR/TE = 8.5/3.9 ms, TI = 1060 ms. Diffusion tensor images (DTI) were acquired with a spin-echo echo planar imaging (SE-EPI) pulse sequence using a 32-direction Stejskal-Tanner diffusion encoding scheme, FOV = 245 × 245 × 150 mm, 60 slices with no interslice gap, spatial resolution = 2.5 mm3, TR/TE = 7639/59 ms, SENSE factor = 2.5, b-values = 0, 1100 s/mm2, dynamic stabilisation and spectral presaturation with inversion recovery (SPIR) fat suppression. Echo-planar imaging (EPI) was used to investigate fluctuations in the blood oxygen level-dependent (BOLD) signal for resting state functional imaging with eyes closed using the following imaging parameters: 30 axial slices, repetition time (TR) / echo time (TE) = 2000 ms/35 ms, flip angle (FA) = 90°, pixel bandwidth = 1780, Hz/Px. Spatial resolution: 2.875 × 2.875 × 4 mm.

### Cortical thickness measurements, medullary volume estimates and brainstem shape analyses

T1-wieghted structural data were pre-processed in for cortical thickness (CT) calculations, medullary volume estimation, brainstem shape profiling and for downstream image registration. CT and medullary volumes were calculated using FreeSurfer version 7.1.0 [8], including automated image segmentation, surface reconstruction and individual CT map output. We defined the bulbar segment of the motor cortex based on the *Brainnetome* atlas [9], which provides functional cortical parcellation based on multimodal imaging data. We refer to labels “A4tl_L” and “A4tl_R” (“tongue/larynx”) of the *Brainnetome* atlas as the “bulbar cortex”. For brainstem segmentation and subsequent medullary volume estimation, we relied on FreeSurfer’s *segmentBS* pipeline [10], which uses a Bayesian algorithm to delineate probabilistic boundaries. To investigate brainstem outline deformations, the *first* pipeline [11] of the FMRIB´s Software Library was implemented [12]; images were skull-stripped and bias-corrected using FSL’s *fsl_anat* pipeline. Subsequently, subcortical structures were segmented using a Bayesian approach and parametrized those labels as surface meshes to run statistical comparisons.

### Appraisal of functional connectivity

Functional data were pre-processed to estimate functional connectivity (FC). FSL’s *feat* [13] was utilized for brain extraction, slice-time correction, and motion correction. Additionally, FSL’s *AROMA* algorithm [14] was implemented to correct for head-motion-related artifacts. Each patient’s pre-processed functional image was linearly co-registered to the native high-resolution structural scan using 6 degrees of freedom (DOFs), and for higher-level group comparisons, non-linearly warped to the MNI152 2 mm standard space (12 DOFs). FC was defined as Fisher *z*-transformed correlation between the mean time courses of the brainstem and the bulbar cortex (separately for the two hemispheres). FC was calculated within *Matlab R2021b* using tools from the CoSMoMVPA toolbox [15].

### Retrieval of structural connectivity metrics

To determine structural connectivity (SC), diffusion-weighted (DW) data were used. Tools from *MRtrix3* (version 3.0.3) [16] were used for pre-processing including noise removal [17], removal of Gibb’s Ringing artifacts [18], motion / eddy current [19] and bias-field corrections [20]. Pre-processed DW images were then aligned to high-resolution T1-weighted data. As fractional anisotropy (FA) is a composite metric of the three eigenvalues (λ_1_, λ_2_, λ_3_), it is histologically non-specific to the underlying white matter pathology and merely offers an overall proxy of white matter integrity. Accordingly, in our study a diffusion-tensor model was fitted to the data to estimate cortico-medullary axial diffusivity (AD) (λ_1_) and radial diffusivity (RD) ((λ_2_ + λ_3_)/2) separately [21]. Tractograms were calculated between the medulla and the right/left bulbar cortices separately in each participant in native space. A probabilistic algorithm was implemented to produce a fixed number of streamlines (n = 5000) between the pairwise ROIs. We then binarized these tractograms to extract the mean AD/RD values per tract in each subject using the previously calculated tensor maps. Multimodal MR data of patients and controls were interrogated both for baseline differences (cross-sectional modelling) as well as differences in progression (longitudinal modelling). Not only disease-associated signatures were explored (*analysis stream 1*) by contrasting all ALS patients to controls, but two specific ALS cohorts were further evaluated. The imaging profile of hexanucleotide repeat expansion carriers in *C9orf72* (“C9 + ”) (*analysis stream 2*) and bulbar asymptomatic (BA) patients (*analysis stream 3*) i.e. C9-, spinal onset patients with a maximum ALSFRS-r bulbar score, were evaluated in dedicated analyses.

### Statistical modelling

The age and education profile of patients and controls were contrasted using Welch two-sample *t*-tests and the ratios of gender and handedness distributions examined by Chi-square testing. All statistical analyses were performed within RStudio (R version 4.2.1) [22]. Multimodal MR data of patients and controls were interrogated both for baseline differences (cross-sectional modelling) as well as differences in progression (longitudinal modelling). Not only disease-associated signatures were explored (analysis stream 1) by contrasting all ALS patients to controls, but two specific ALS cohorts were further evaluated. The imaging profile of hexanucleotide repeat expansion carriers in C9orf72 (“C9 + ”) (analysis stream 2) and sporadic bulbar asymptomatic (BA) patients (analysis stream 3) i.e. C9-, spinal onset patients with a maximum ALSFRS-r bulbar score, were evaluated in dedicated analyses. Each of these streams were explored cross-sectionally and longitudinal, whereby the cross-sectional model comprised a simple linear model for analysis stream 1 and a one-way analysis of variance (ANOVA) for analysis streams 2 and 3 (given that these contrasted three groups). Age, sex and handedness were included as covariates and volumetric analyses were also adjusted for total intracranial volume (TIV). Additionally, post-hoc Tukey’s Honest Significance Difference (HSD) testing was implemented to explore pairwise differences between groups. To evaluate brainstem outline alterations at baseline, non-parametric statistical comparisons were implemented between all ALS patients and HC (analysis stream 1), C9 + vs. C9- ALS patients (analysis stream 2) and BA vs. bulbar symptomatic (BS) C9-patients (analysis stream 3). For each comparison, we applied FSL’s randomise [23] algorithm with 5000 permutations and 2D-optimized threshold-free cluster enhancement (TFCE), using a two-sample t-test design covarying for age, gender and handedness. Since two-sided testing was performed, significance threshold was set to *p* ≤ 0.025. Longitudinal changes were evaluated in linear mixed effects models, where Time (i.e. imaging timepoint) was considered as a random effect and the subjects as fixed effects. In “analysis stream 1”, the interaction between Time and Group, in “analysis stream 2” progression differences between C9 + vs. C9–/HC and in “analysis stream 3”, differences between C9- BA and BS patients were evaluated. Similar to the cross-sectional analyses at baseline, age, sex and handedness were included as covariates. To test the discriminatory potential of specific imaging metrics in our panel of radiological indices, we ran Receiver Operator Characteristics (ROC) analyses between all ALS patients and controls and tested the likelihood that a given area under the curve (AUC) value differed from 0.5. Given the relentless clinical progression in ALS, we hypothesized that AUCs may increase over time, therefore we included only patients who had a baseline and a 12-month follow-up scan (*N* = 67 vs. 147 HC) and computed ROC and corresponding AUC values for each imaging metrics at the two time-points 12-months apart. To test the hypothesis of increasing AUCs, we ran a one-sided paired t-test between the AUC of all 9 analysed imaging metrics at baseline and follow-up. The ROC/AUC analyses were carried out within RStudio using the pROC package [24]. Clinico-radiological associations were explored for bulbar motor (ALSFRS-r) scores. Cross-sectional associations with bulbar subscores at baseline were tested in a linear model incorporating the relevant covariates. Longitudinal associations were investigated based on the change in ALSFRS-r bulbar subscores as the independent, and the difference between the given MRI metric as dependent variables, correcting for age, gender and handedness.

### Data availability

Group-level outputs and additional information on data processing can be requested from the corresponding author. Individual-patient clinical, genetic and imaging data cannot be transferred due to institutional and departmental policies.

## Results

### Demographics

Demographic a clinical data are presented in Table 1. Welch two-sample *t*-test revealed no significant age differences between ALS patients and controls (*t*(241.6) =  – 0.58, *p* = 0.57). Chi-square test captured sex differences (*X*^*2*^ (1, *N* = 306) = 6.06, *p* = 0.01), but no differences in handedness (*X*^*2*^ (1, *N* = 306) = 0.08, *p* = 0.78). All patients tested negative for a panel of protein-altering, exonic or splice-site variants in 32 genes linked to ALS.

### Progressive cortico-medullary disconnection is specific for ALS

**Fig. 2:**
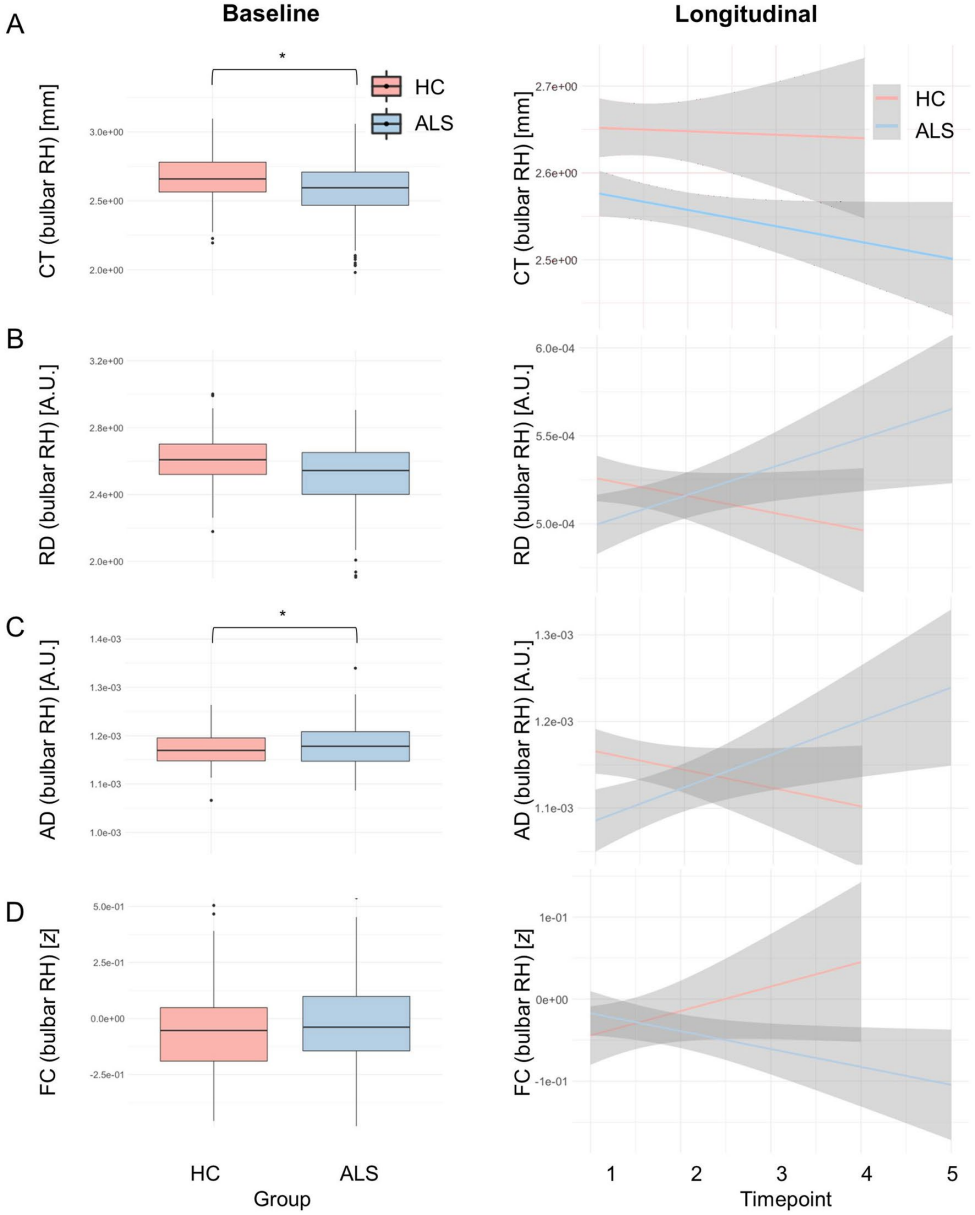
Analysis stream 1: Disease-associated imaging traits in unselected (all) ALS patients. Cross-sectional (left panel) and longitudinal (right panel) imaging alterations in unstratified ALS patients with reference to healthy controls. **A** Cortical thickness of the right bulbar cortex and **C** cortico-medullary axial diffusivity differentiates ALS from controls at baseline. Connectivity metrics reveal progressive cortex-brainstem disconnection based on **B** radial diffusivity (RD), **C** axial diffusivity (AD) and **D** functional connectivity (FC). Detailed descriptive statistics are presented in Table 2. Boxplots represent medians $\pm$ 1 quartile, whiskers denote a data range spanning the median $\pm 1.58 \times IQR\sqrt{n}$, dots represent outliers, whereby IQR is the interquartile range. * denotes statistical significance of unpaired *t*-tests, thresholded at *p* ≤ 0.05. *AD* axial diffusivity, *ALS* patients with amyotrophic lateral sclerosis, *CT* cortical thickness, *FC* functional connectivity, *HC* Healthy control, *IQR* interquartile range, *RD* radial diffusivity

**Fig. 3:**
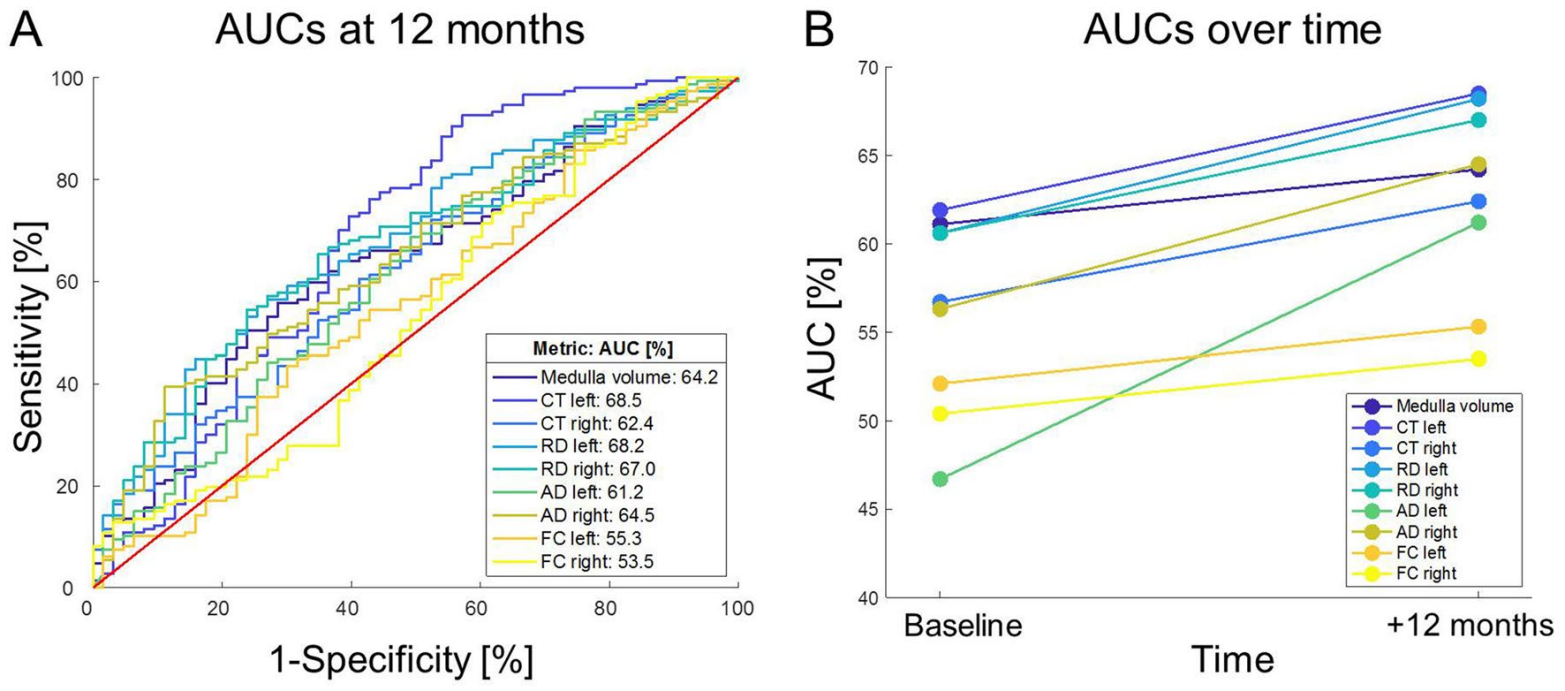
The discriminatory hierarchy of bulbar neuroimaging markers between patients with ALS and healthy controls based on area under the curve (AUC) values their Receiver Operator Characteristics (ROC) analyses (**A**). AUC values of all bulbar metrics increased over a 1-year follow-up (**B**) (*p* < 0.001)

**Fig. 4:**
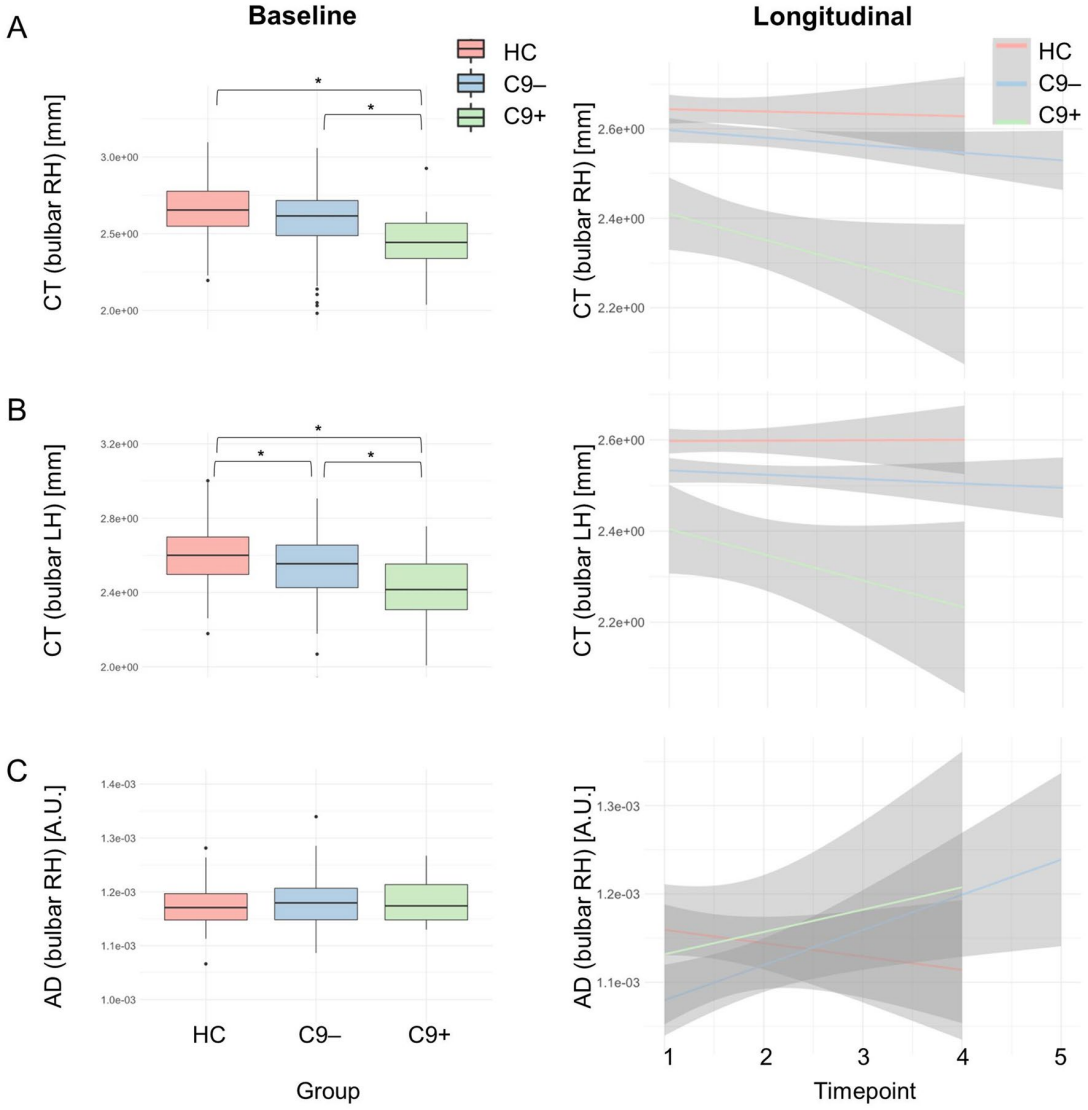
Analysis stream 2: the evaluation of bulbar imaging indices in GGGGCC hexanucleotide repeat expansion carriers in *C9orf72*. Cross-sectional patterns (left panel) and longitudinal changes (right panel) were evaluated at cortical level, in cortico-medullary connectivity and in the brainstem. Detailed descriptive statistics are presented in Table 2. Boxplots represent medians $\pm$ 1 quartile, whiskers denote a data range spanning the median $\pm 1.58 \times IQR\sqrt{n}$, dots represent outliers, whereby IQR is the interquartile range. * denotes statistical significance of unpaired *t*-tests, thresholded at *p* ≤ 0.05. *AD* axial diffusivity, *ALS* patients with amyotrophic lateral sclerosis, *C9 +*  ALS patients with GGGGCC hexanucleotide repeat expansion in *C9orf72*, *C9*- ALS patients without GGGGCC hexanucleotide repeat expansion in *C9orf72*, *CT* cortical thickness, *FC* functional connectivity, *HC* Healthy control, *IQR* interquartile range, *RD* radial diffusivity

**Fig. 5:**
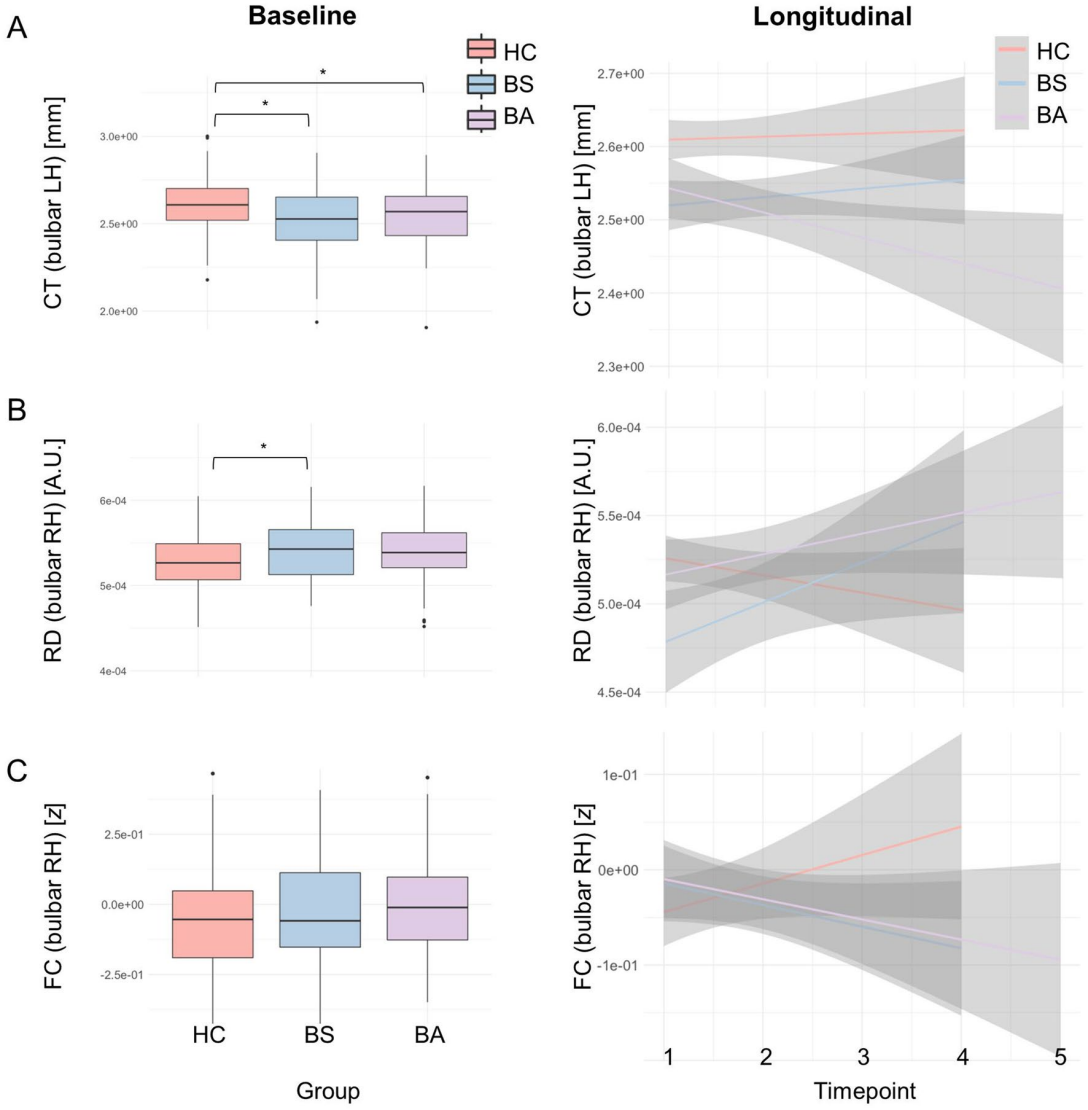
Analysis stream 3: the evaluation of “bulbar asymptomatic” patients i.e. sporadic patients with ALS with spinal onset disease without bulbar symptoms, without a family history of ALS, who tested negative for *C9orf72*. Cross-sectional patterns (left panel) and longitudinal changes (right panel) were evaluated at cortical level, in cortico-medullary connectivity and in the brainstem. Patients who are asymptomatic from a bulbar disability perspective, already exhibit cortical thickness reductions at baseline. Detailed descriptive statistics are presented in Table 2. Boxplots represent medians $\pm$ 1 quartile, whiskers denote a data range spanning the median $\pm 1.58 \times IQR\sqrt{n}$, dots represent outliers, whereby IQR is the interquartile range. *denotes statistical significance of unpaired t-tests, thresholded at *p* ≤ 0.05. *AD* axial diffusivity, *ALS* patients with amyotrophic lateral sclerosis, *BA* “bulbar asymptomatic” *C9*- patients without bulbar symptoms *BS* C9- patients with bulbar symptoms, *C9* ALS patients without GGGGCC hexanucleotide repeat expansion in *C9orf72*, *CT* cortical thickness, *FC* functional connectivity, *HC* Healthy control, *IQR* interquartile range, *RD* radial diffusivity

The main statistical outputs of neuroimaging analyses are presented in Table 2 and illustrated in Figs. 2, 3, 4, 5. Considering all ALS patients (Fig. 2), more rapid cortico-medullary disconnection was identified in patients compared to controls in the right hemisphere. The interaction effects (*Group x Time*) in the longitudinal models revealed RD increase [*t*(237) = 2.030, *p* = 0.044], AD increase [*t*(237) = 2.210, *p* = 0.028] and FC decline over time [*t*(237) =  – 2.187, *p* = 0.030]). Interestingly, the CT of the bilateral bulbar cortex – while not exhibiting progressive change – was significantly thinner in patients at baseline (both RH/LH: *p* < 0.001). No medullary volume (MV) or brainstem shape (BrS) differences were detected between the study groups.

**Table 2:** Cross-sectional and longitudinal outputs for the panel of imaging metrics

	Cross-sectional (Main effect: Group)						Longitudinal (Interaction: Time x Group)					
	Right hemisphere			Left hemisphere			Right hemisphere			Left hemisphere		
	Esti-mate	*t */ F-value	*p*-value	esti-mate	*t */ F-value	*p*-value	esti-mate	*t */ F-value	*p*-value	esti-mate	*t*-value	*p*-value
ALS vs. HC												
Vol	– 6.75e-5	– 1.505	0.133	*n.a. *	*n.a. *	*n.a. *	– 3.20e-5	– 1.263	0.208	*n.a.*	*n.a. *	*n.a. *
CT	– 0.076	– 3.375	8.34e-4*	– 0.088	– 4.315	<.001*	– 0.022	– 1.686	0.093	– 6.20e-3	– 0.639	0.524
RD	– 2.26e-5	– 1.376	0.170	– 7.63e-6	– 0.454	0.651	2.58e-5	2.030	0.044*	2.07e-5	1.626	0.105
AD	– 7.58e-5	– 2.151	0.032*	– 4.74e-5	– 1.320	0.188	6.01e-5	2.210	0.028*	4.35e-5	1.567	0.118
FC	0.026	1.035	0.302	0.005	0.185	0.853	– 0.048	– 2.187	0.030*	0.004	0.174	0.862
C9+ vs. C9-/HC												
Vol	9.40e-7	3.427	0.034*	*n.a. *	*n.a. *	*n.a. *	5.82e-6	0.161	0.872	*n.a. *	*n.a. *	*n.a. *
CT	0.920	14.11	<.001*	0.709	12.85	<.001*	– 0.029	– 2.040	0.042*	– 0.044	– 3.505	<.001*
RD	9.30e-8	2.533	**0.081**^**+**^	4.40e-8	1.144	0.320	– 1.56e-5	– 0.746	0.456	2.04e-8	9.75e-4	0.999
AD	5.57e-7	3.276	0.039*	3.17e-7	1.794	0.168	– 2.72e-5	– 0.597	0.551	– 1.50e-5	– 0.325	0.745
FC	0.050	0.599	0.550	0.006	0.079	0.924	– 0.003	– 0.074	0.941	– 0.024	– 0.651	0.515
BA/BS vs. HC* (C9- patients only)*												
Vol	1.03e-6	3.813	0.023*	*n.a. *	*n.a. *	*n.a. *	3.80e-5	1.543	0.124	*n.a. *	*n.a. *	*n.a. *
CT	0.300	4.707	0.010*	0.433	8.233	<.001*	0.0172	1.654	**0.100+**	1.03e-4	0.011	0.991
RD	1.65e-7	4.460	0.012*	1.18e-7	3.042	0.049*	– 3.11e-5	– 2.183	0.030*	– 3.97e-6	– 0.318	**0.095+**
AD	1.03e-6	6.074	0.003*	8.01e-7	4.500	0.012*	– 6.96e-5	– 2.304	0.022*	– 5.45-e5	– 1.778	**0.077+**
FC	0.076	0.905	0.406	0.043	0.507	0.603	0.048	1.961	**0.051+**	– 0.021	– 0.862	0.390
MRI-clinical correlations (ALSFRS-r bulbar score)												
Vol	266.22	0.361	0.7193	*n.a. *	*n.a. *	*n.a. *	3.59e-05	0.809	0.421	*n.a. *	*n.a. *	*n.a. *
CT	– 0.638	– 0.565	0.574	– 0.567	0.508	0.613	– 5.12e-4	– 0.027	0.978	– 0.012	– 0.949	0.346
RD	1.58e3	0.868	0.389	8.18e2	0.454	0.651	– 3.14e-5	– 1.286	0.202	– 1.58e-5	– 0.578	0.565
AD	689.01	0.808	0.422	711.43	0.843	0.402	– 5.17e-5	– 0.974	0.333	– 3.10e-5	– 0.541	0.590
FC	1.032	0.883	0.380	-0.324	– 0.251	0.802	9.88e-4	0.022	0.982	5.57e-3	– 0.119	0.906
Receiver operating characteristics (ROC)												
ROI	AUC at baseline	AUC at 12 months										
Medulla volume	0.611	0.642										
CT right	0.567	0.624										
CT left	0.619	0.685										
RD right	0.606	0.670										
RD left	0.606	0.682										
AD right	0.563	0.645										
AD left	0.467	0.612										
FC left	0.504	0.535										
FC right	0.521	0.553										

For unilateral structures, such as the medulla, relevant values are presented in the “left hemisphere” columns. For the cross-sectional comparisons “**ALS vs. HC**” and the “**MRI-clinical correlations**”, *t*-tests are reported; for the cross-sectional comparisons “**C9 + vs. C9/HC**” and “**BA/BS vs. HC**” we *F*-tests (analyses of variance) are reported. Where the *F*-test was significant for cross-sectional comparisons, we performed pairwise post-hoc tests. Significant post-hoc differences are indicated in Figs. 4 and 5 using asterisks. *AD* axial diffusivity, *ALS* amyotrophic lateral sclerosis, *AUC* area under the curve, *BA* bulbar asymptomatic, defined as C9orf72 negative with spinal onset disease an no bulbar symptom at baseline, *BS* bulbar symptomatic, defined as C9orf72 negative patient with bulbar symptoms at baseline, *C9 +*  hexanucleotide repeat expansion carrier in C9orf72, *C9-* tested negative for hexanucleotide repeat expansions in C9orf72, *CT* cortical thickness, *FC* functional connectivity, *HC* healthy control, *MRI* magnetic resonance imaging, *n.a.* not applicable, *RD* radial diffusivity, *ROC* receiver operating characteristics, *Vol* medullary volume, ^+^approaching significace at an alpha-level of *p* ≤ 0.10, *significant at an alpha-level of *p* ≤ 0.05

### The discriminatory profile of bulbar neuroimaging metrics and their longitudinal trajectory

Our ROC analyses and the derived AUC values confirmed the discriminatory potential of nearly all analysed neuroimaging metrics. At baseline, medullary volume showed the highest discriminatory power for distinguishing patients from controls (AUC = 0.611, *p* = 0.006, Fig. 3A), followed by CT (LH: AUC = 0.619, RH: AUC = 0.567) and RD (LH: AUC = 0.606, RH: AUC = 0.606), all exhibiting high AUCs. Notably, the AUCs of all analysed metrics increased over time (*t*(8) = 5.431, *p* < 0.001; Fig. 3B).

### Cortical thickness decreases more rapidly in hexanucleotide repeat expansion carriers

Following patient stratification by *C9orf72* status (Fig. 4), CT was identified as an important moderator of progression: bulbar cortex CT loss in C9 + patients was more rapid than in C9- or HC group [RH: *t*(236) =  – 2.040, *p* = 0.042; LH: *t*(236) =   – 3.505, *p* < 0.001; based on the *Group x Time* interaction effect with C9 + as a reference group]. Moreover, ANOVA confirmed significant bilateral CT differences already at baseline [RH: *F*(2298) = 14.11, *p* < 0.001; LH: F(2298) =  – 2.040, *p* = 0.052], whereas post-hoc Tukey HSD testing revealed that C9 + patients were the main drivers of this effect [RH: thinner CT of C9 + vs. C9–: *p* < 0.001; LH: thinner CT of C9 + vs. C9–: *p* < 0.001]. The comparison of AD also revealed a genotype-effect [RH: F(2298) = 14.11, *p* = 0.0391], but pairwise group differences could not be confirmed by post-hoc Tukey HSD testing. Data visualization (Fig. 4C) reveals a tendency for increased AD in both ALS groups vs. HC. Brainstem volumes and brainstem shape profiles were not modulated by genetic status.

### Bulbar asymptomatic patients exhibit multi-level presymptomatic alterations

Spinal onset C9- patients without bulbar manifestations (“bulbar asymptomatic”- BA) exhibit marked radiological changes at baseline (Fig. 5) based on four neuroimaging metrics: (1) medullary volume [*F*(2276) = 1.03e-6, *p* = 0.023]; (2) bilateral CT [RH: *F*(2, 276) = 0.300, *p* = 0.010; LH: *F*(2, 276) = 0.433, *p* < 0.001], (3) bilateral cortico-medullary RD [RH: *F*(2276) = 1.65e-7, *p* = 0.012; LH: *F*(2276) = 1.18e-7, *p* = 0.049]] and (4) bilateral cortico-medullary AD [RH: *F*(2276) = 1.03e-6, *p* = 0.003; LH: *F*(2276) = 8.01e-7, *p* = 0.012]. Post-hoc Tukey HSD tests confirmed left-hemispheric CT reduction even in bulbar asymptomatic patients compared to controls (*p* = 0.017). From a cortico-medullary connectivity perspective, BA patients did not differ from BS patients in right-hemispheric RD (*p* = 0.061) and with regards to left-hemispheric RD, BA patients exhibited higher values compared to BS (*p* = 0.044). No post-hoc differences were detected in medullary volumes or FC in pairwise comparisons. Longitudinally, right-hemispheric structural connectivity – both RD and AD – deteriorated in BA similarly to BS with reference to controls [RD: *t*(219) =  – 2.183, *p* = 0.030; AD: *t*(219) =  – 2.304, *p* = 0.022. Moreover, right-hemispheric FC tended to decrease in BA similarly to BS vs. controls [FC: *t*(219) = 1.961, *p* = 0.051]. These observations suggest that considerable disease burden can be ascertained in anatomical regions associated with bulbar function before bulbar disability develops, both at a cortical level and also from a cortico-medullary connectivity perspective. No presymptomatic shape alterations were detected in the BA group.

### Dissociation between bulbar disability scores and imaging metrics

No direct correlations were identified between bulbar ALSFRS-r subscores and any of the radiological integrity metrics (CT, AD, RD, FC, MV, BrS), neither cross-sectionally nor longitudinally. Output statistics are summarized for all neuroimaging metrics in Table 2 to demonstrate the dissociation between motor disability and cerebral imaging measures.

## Discussion

We have evaluated the integrity of anatomical structures involved in bulbar function in a large cohort of genetically and clinically characterised patients in a longitudinal imaging study using a standardised imaging protocol. Our analyses revealed progressive structural and functional disconnection between the motor cortex and the brainstem over time. Cortical thickness reduction was an early feature on cross-sectional analyses with limited further progression on longitudinal follow-up. Hexanucleotide repeat carriers exhibited lower brainstem volumes, lower cortico-medullary structural connectivity and faster cortical thinning. While brainstem and corticobulbar tract involvement are well established post mortem, these brain regions are challenging to quantitatively evaluate in vivo. Despite its considerable clinical implications, the substrate of bulbar impairment in ALS is poorly characterised at present. ALS is associated with progressive brainstem–cortex disconnection which is particularly rapid in *C9orf72* hexanucleotide repeat carriers. The systematic analysis of a large panel of imaging metrics demonstrates that some metrics show discriminatory potential between patients and controls at baseline, but exhibit limited change over time; these may be ideally suited for diagnostic applications. Conversely, other metrics may not readily discriminate patients from controls at baseline, but capture subtle changes over very short follow-up periods, making them particularly useful for monitoring applications. Our study also highlights that despite preserved bulbar function at the time of MR imaging, significant degenerative change can already be observed in the relevant brains regions. Disease burden in ALS is best evaluated in by robust multimodal studies and academic studies have the potential to inform the design of streamlined pharmacological trial protocols. The combination of a fast-acquisition 3D T1-weighted and a diffusion tensor imaging protocol offer ample biomarker potential both for clinical trial applications obviating the need for complex fMRI analyses.

The targeted evaluation of a cohort of hexanucleotide expansion carriers in *C9orf72* confirmed the unique clinical and radiological attributes of this genotype. The radiological signature of *C9orf72* is classically associated with marked frontotemporal change and resulting cognitive dysfunction. It is increasingly clear however that marked frontotemporal change in ALS is not unique to *C9orf72* [25] and that patients with this genotype may also have distinguishing cerebellar, spinal cord and other extra-motor changes [7, 26]. Our finding of a more rapid neurodegenerative process in this cohort is well in line with both clinical observations and other neuroradiology studies [27].

Presymptomatic changes are of huge interest in ALS and considerable pathological change has been consistently demonstrated in mutation carriers [26, 28, 29]. Pioneering studies of presymptomatic disease-burden not only offer a window on incipient changes, the sequential involvement of anatomical structures and propagation patterns for academic research [4, 30], but from a pragmatic, clinical view point, they may inform the ideal timing of future pharmacological interventions [31, 32]. Familial cases and carriers of pathogenic mutations however only represent a small minority of patients with ALS. It is likely that “sporadic” patients also accrue disease burden long before symptom onset and certainly well before the diagnosis is confirmed. Radiological observations from presymptomatic *SOD1* and *C9orf72*, while conceptually important, may not be directly transferrable to “sporadic” ALS due to their distinctive anatomical signatures and differing progression rates. Accordingly, the presymptomatic phase of “sporadic ALS” remains notoriously elusive and we currently merely rely on indirect insights derived from gene carriers. Recent presymptomatic studies describe slowly progressive neurodegenerative changes decades before symptom onset, and raise the possibility of developmental factors [33]. It has been speculated that ample degenerative change has to take place for symptom manifestation, and that there may be a certain threshold when compensatory circuits and inherent functional redundancy are exploited. Analogous to the concept of cognitive reserve, terms such as “motor reserve” have been coined [34], but not compellingly demonstrated. As we have shown in this study, it may be possible to study symptomatic cohorts of patients who are asymptomatic in a specific clinical domain, in our case bulbar function, and appraise the integrity of the relevant structures involved in that specific function. Our study indicates, that despite preserved bulbar function in sporadic patients with ALS, significant degenerative change can already be observed in relevant brains regions.

One of the many roles of academic neuroimaging studies is to critically appraise the practical utility of a spectrum of radiology metrics to inform the design of streamlined clinical trial applications. While MRS, QSM, rsFMRI, NODDI, spinal cord metrics, etc. all offer invaluable academic insights [26, 35–41], they are not routinely implemented in the clinical setting. As demonstrated by this study, a high-resolution structural dataset can be flexibly interrogated in a multitude of pipelines and a multitude of open-source software libraries are available for transparent data interpretation. Similar to the versatility of structural data, DWI/DTI data can be meaningfully interrogated by tractography, tract-based statistical approaches or in connectomic models [42–44]. Our data indicate that contrary to previous reports [45], vertex analyses have relatively little to offer at a brainstem level; brainstem outline alterations merely reflect overall shape deformations and may not meaningfully capture focal pathology in relevant structures such as cranial nerve nuclei or descending corticospinal tract degeneration. Similarly, the assessment of medullary volumes revealed no disease-associated or genotype-specific signatures either cross-sectionally or longitudinally. The absence of medullary volume reduction is not surprising given the selective and focal involvement of specific brainstem structures instead of a more global process. Another practical aspect of protocol development is ease of data harmonisation [46, 47] which is particularly pertinent to low-incidence conditions such as ALS requiring multi-site collaborations for sufficient statistical power. Clinical trials are also invariably multi-site, necessitating stringent protocol harmonisation. In our study, the discriminatory potential of bulbar imaging measures between patients and controls were evaluated by receiver operating characteristic analyses. While AUC values did not reach 0.7 which is commonly regarded as a cut-off for excellent discrimination, at 12-month follow-up, most of the AUC values were over 0.6 suggestive of acceptable discrimination. Machine-learning frameworks have been increasing applied to large ALS datasets [48, 49] and feature importance analyses have invariably highlighted the role of cortical grey and white matter diffusivity measures [36, 50–55]. To demonstrate the diagnostic utility of such models however, classification models need to be tested and validated on early-stage patients or patients soon after their diagnoses [56]. The accurate categorisation of late-stage or patients with considerable disability says relatively little about the practical utility of a particular model. This notion is demonstrated by the AUC profile of our panel of bulbar metrics which all increase over time (Table 2).

Patients with pseudobulbar affect (PBA) experience sudden tearing or laughing in response to minimal emotional stimuli. Patients with PBA are well aware of their exaggerated reactions and often choose to avoid social interactions [57]. In recognition of the considerable quality of life implications of PBA, a multitude of pharmacological trials have been conducted recently [58]. While the classical conceptualisation of pseudobulbar affect centres on the loss of corticobulbar inhibition i.e. cortico-medullary disconnection, more recent PBA studies highlight the role of impaired cerebellar gating as well as extra-motor control network dysfunction [57, 59–63]. We also note that an interaction between cognitive manifestations and bulbar impairment has been consistently suggested by epidemiology, neuroimaging and neuropsychology studies [30, 64–67] and the more detailed assessment of descending frontopontine, temporopontine and parietopontine fibres may reveal additional insights. Finally, it is noteworthy that corticobulbar tract degeneration and bulbar dysfunction are not unique to ALS, but also commonly observed in primary lateral sclerosis (PLS) [68–70]. While PLS typically manifest with lower limb spasticity initially, spastic dysarthria and pseudobulbar affect commonly ensue over the course of the disease [71, 72]. The development of non-invasive cortico-medullary connectivity measures may therefore be relevant to other neurodegenerative conditions, and more broadly, to other conditions where pseudobulbar dysfunction is an important feature, such as multiple sclerosis (MS).

This study is not without limitations. We acknowledge the scarce follow-up data on the healthy control cohort, which were acquired to account for healthy ageing, but more complete normative data sets would be desirable for accurate longitudinal modelling. Moreover, the lack of post mortem data precludes the histopathological validation of our radiological findings. Notwithstanding these limitations, our data demonstrates progressive cortex-brainstem disconnection as a unifying feature of ALS biology.

## Conclusions

ALS is associated with progressive brainstem–cortex disconnection which is particularly rapid in *C9orf72* hexanucleotide repeat expansion carriers. Imaging indices differ considerably in their detection sensitivity and ability to track progressive pathological changes. Disease burden in ALS is therefore best evaluated by a panel of complementary imaging markers. Academic studies have the potential to inform the design of streamlined clinical and future pharmacological trial protocols.

## Authorship contribution

The manuscript was drafted by MT, ELT, WFS, PB. Study conceptualisation: MT, PB. Clinical assessments: RC, OH, PB. MR data processing and analyses: MT, ELT, PB. Genetics analyses: RLMcL, MAD, AV, JCH.

## Supplementary Information

Below is the link to the electronic supplementary material.

Supplementary file1 (PDF 273 KB)

## Abbreviations

**AD**: Axial diffusivity
**ALS**: Amyotrophic lateral sclerosis
**ALSFRS-r**: Revised amyotrophic lateral sclerosis functional rating scale
**ANOVA**: Analysis of variance
**AUC**: Area under the curve
**BA**: “Bulbar asymptomatic”- patients with spinal onset disease without bulbar symptoms who tested negative for *C9orf72* repeat expansions
**BrS**: Brainstem shape
**BOLD**: Blood-oxygen-level-dependent (BOLD) signal
**BS**: “Bulbar symptomatic”- patients with bulbar symptoms
**C9**** + **: ALS patients with GGGGCC hexanucleotide repeat expansion in C9orf72
**C9-**: ALS patients without GGGGCC hexanucleotide repeat expansion in C9orf72
***C9orf72***: Chromosome 9 open reading frame 72
**CBT**: Corticobulbar tract
**CST**: Corticospinal tract
**CT**: Cortical thickness
**DC**: Disease control
**DTI**: Diffusion tensor imaging
**EMG**: Electromyography
**EMM**: Estimated marginal mean
**EPI**: Echo-planar imaging
**FC**: Functional connectivity
**FLAIR**: Fluid-attenuated inversion recovery
**fMRI**: Functional MRI
**FOV**: Field of view
**FSL**: FMRIB´s Software Library
**FTD**: Frontotemporal dementia
**FWE**: Familywise error
**GM**: Grey matter
**HARDI**: High angular resolution diffusion imaging
**HC**: Healthy control
**IR-SPGR**: Inversion recovery prepared spoiled gradient recalled echo
**IQR**: Interquartile range
**IR-TSE**: Inversion recovery turbo spin echo sequence
**LH**: Left hemisphere
**LMN**: Lower motor neuron
**Lt**: Left
**ML**: Machine-learning
**MND**: Motor neuron disease
**MNI152**: Montreal neurological Institute 152 standard space
**MRS**: MR spectroscopy
**MS**: Multiple sclerosis
**MUNE**: Motor unit number estimation
**MUNIX**: Motor unit number index
**MV**: Medullary volume
**NISALS**: Neuroimaging Society in ALS
**NODDI**: Neurite orientation dispersion and density imaging
**PBA**: Pseudobulbar affect
**PLS**: Primary lateral sclerosis
**PMC**: Primary motor cortex
**QC**: Quality control
**QSM**: Quantitative susceptibility mapping
**RH**: Right hemisphere
**RD**: Radial diffusivity
**ROC**: Receiver operator characteristic curve
**ROI**: Region of interest
**rsFMRI**: Resting-state functional MRI
**Rt**: Right
**SBMA**: Spinal-bulbar muscular atrophy (SBMA)
**SC**: Structural connectivity
**SD**: Standard deviation
**SE-EPI**: Spin-echo echo planar imaging
**SENSE**: Sensitivity Encoding
**SMA**: Spinal muscular atrophy
**SPIR**: Spectral presaturation with inversion recovery
**T**: Tesla
**T1w**: T1-weighted imaging
**TE**: Echo time
**TFCE**: Threshold-free cluster enhancement
**TI**: Inversion time
**TIV**: Total intracranial volume
**TR**: Repetition time
**Tukey HSD tests**: Tukey's Honest Significant Difference test
**UMN**: Upper motor neuron
**WM**: White matter
